# Changes in the profile of newly HIV-diagnosed men who have sex with men, Madrid, 2014 to 2019

**DOI:** 10.2807/1560-7917.ES.2021.26.47.2001501

**Published:** 2021-11-25

**Authors:** Oskar Ayerdi Aguirrebengoa, Mar Vera Garcia, Teresa Puerta López, Petunia Clavo Escribano, Juan Ballesteros Martín, Clara Lejarrag Cañas, Enrique Fuentes Ferrer, Montserrat Raposo Utrilla, Vicente Estrada Perez, Jorge Del Romero Guerrero, Carmen Rodríguez Martín, Sandoval Study Group, Sandoval Study Group, Estefania Hurtado, Monica García, Natividad Jerez, Sonsoles Del Corral, Marta Ruiz, Marta Gonzalvo, Elena Gutiérrez, Cristina Quirós, Víctor Rodríguez.

**Affiliations:** 1 Centro Sanitario Sandoval, Hospital Clínico San Carlos, IdISSC, Madrid, Spain; 2 Universidad Complutense de Madrid, Madrid, Spain; 3 Servicio de Medicina Preventiva, Insituto de investigación Sanitaria San Carlos, Universidad Alfonso X el Sabio, Madrid, Spain; 4 Unidad de Infecciosas, Medicina Interna, Hospital San Carlos, IdISSC, Madrid, Spain

**Keywords:** HIV, seroconvertors, epidemiology, prevention, chemsex

## Abstract

**Introduction:**

Knowing the factors associated with HIV transmission is necessary in order to design preventive programmes tailored to the epidemiological situation in each region and population.

**Aim:**

Our objective was to study the sociodemographic, clinical and behavioural characteristics of men who have sex with men (MSM) who were newly diagnosed with HIV infection.

**Methods:**

We carried out an observational, descriptive, study on all MSM newly diagnosed with HIV infection in one clinic for sexually transmitted infections (STI) and HIV clinic in Madrid between 2014 and 2019. Information on sociodemographic, clinical, and behavioural characteristics of participants per year of diagnosis was collected.

**Results:**

We detected a total of 1,398 people with HIV infection, 253 of whom were recent seroconverters (rSCV) with a median duration of documented seroconversion of 6 months. From the total, 97.9% infections were sexually transmitted and 2.1% involved injected drugs, i.e. slam practices. The average age was 32.9 years (range: 15.6–74.9), 51.8% were Spanish and 40% Latin American. These diagnoses decreased in Spanish people and increased in Latin Americans during the study period. Of the rSCV, 73.9% had condomless sex under the influence of drugs and 28.9% participated in chemsex sessions. Apps were used by 92.6% rSCV for sexual encounters and 70.4% of them attributed HIV transmission to their use.

**Conclusions:**

Combination of HIV prevention strategies, as pre-exposure prophylaxis, should be reinforced among young MSM, especially those born in Latin America, those who use drugs for sex, and those who use apps in search of sexual contacts.

## Introduction

Since the start of the AIDS epidemic, the interest in knowing the factors associated with the transmission of infection of the human immunodeficiency virus (HIV) has been constant in order to establish up-to-date prevention strategies aimed particularly at the most affected groups [1].

Numerous studies show that HIV transmission is higher in infected subjects who are unaware of their serostatus [2]. Thus, early diagnosis and highly active antiretroviral therapy (HAART) are essential tools for prevention. In 1987, the sale of the first drug, Zidovudine (AZT), was approved, but it was not until 1996 that the greater efficacy of combination ART changed the history of the infection [3,4]. Since then, advances in ART have dramatically improved the survival and quality of life of infected people [5]. Several studies have confirmed that seropositive patients with undetectable viral load under ART did not transmit the infection to their sexual partners [6,7]. Therefore, the reduction of the time between the transmission of HIV and the diagnosis and treatment of the infection is a priority in all prevention programmes for this disease. Several preventive measures have been used against HIV, such as the promotion of consistent condom use, sex education, screening for sexually transmitted infections (STI) and HIV, post-exposure prophylaxis (PEP) or early diagnosis and immediate ART, which is probably the measure that has had the greatest impact in recent years. In 2016, another preventive measure known as pre-exposure prophylaxis (PrEP) was approved in Europe, recommended by different health agencies and scientific societies such as the United States (US) Centers for Disease Control and Prevention (CDC), the World Health Organization (WHO), the European AIDS Clinical Society (EACS) or the Spanish AIDS Study Group (GeSIDA) [8-13]. In November 2019, the Ministry of Health announced the funding of PrEP in Spain as an additional measure of prevention against HIV within the National Health System [14]. According to UNAIDS data, there has been a 16% reduction in new HIV diagnoses globally, from 2.1 million in 2010 to 1.7 million in 2018 [15]. In some high-income countries, the implementation of preventive strategies in a combined manner and directed at target populations has been associated with an important decrease in new cases of HIV, even in men who have sex with men (MSM) for the first time in years [16-18].

In 2018, 26,164 new cases of HIV infection were reported in Europe, a rate of 5.6 per 100,000 people/year [19]. According to the Sistema de Información sobre nuevos diagnósticos de VIH (SINIVIH) of the Ministry of Health, 3,244 new cases were detected in Spain in 2018, a rate of 8.65 per 100,000 inhabitants, the majority among MSM; the usual mode of transmission was sexual [20].

The objective of this work was to describe the sociodemographic, clinical and behavioural characteristics of MSM newly diagnosed with HIV infection between 2014 and 2019 in a reference centre for STI/HIV in Madrid and to analyse changes during that period.

## Methods

We carried out an observational, descriptive study in a reference clinic for STI/HIV in Madrid between 2014 and 2019. That centre offers universal care without administrative barriers, attends to more than 30,000 consultations a year and is located in the heart of the city. All MSM with new HIV diagnosis during the 6-year period were invited by the health professional, on the same day or close to the diagnosis, to participate in the study; they were also offered linkage for immediate ART and STI screening. The treating physician compiled a complete clinical history of each patient based on the information on sociodemographic, clinical and behavioural characteristics collected STI/HIV clinic. The collected variables are described in Table 1.

**Table 1:** Study variables collected for men who have sex with men newly diagnosed with HIV, Madrid, 2014 to 2019

Variables	
Mode of transmission	Sexual; people who inject drugs
Age	≤ 19; 20–29; 30–39; 40–49; 50–59; ≥ 60 years
Place of birth	Spain; Latin America; Other
CD4^+^ T-lymphocyte count	Advanced disease < 200 cells/μL; late diagnosis 200–350 cells/μL; 351–500 cells/μL; early diagnosis > 500 cells/μL
Plasma HIV viral load	< 37 cop/mL; 37–1,000 cop/mL; 1,001–10,000 cop/mL; 10,001–50,000 cop/mL; 50,001–100,000 cop/mL; > 100,000 cop/mL
Prior negative results at the time of HIV diagnosis	Yes/no
HIV recent seroconverters^a^	Yes/no
History of other STI^b^	Yes/no
Concomitant STI^b^ at the time of HIV diagnosis	Yes/no
Age of sexual debut	< 15; 15–18; 19–25; > 25 years
Number of sexual partners in the year before HIV diagnosis	0–5; 6–25; 26–100; > 100
Number of sexual partners during lifetime	1–10; 11–100; 101–1,000; > 1,000
Consistent condom use according to sexual activity in the year before HIV diagnosis	Orogenital, vaginal or anal sex
Sex worker	Yes/no
Use of PEP and PrEP	Yes/no
Use of drugs for sex in the year before the diagnosis of HIV	Alcohol 'in excess', cannabis, cocaine, poppers, ketamine, GHB, ecstasy, mephedrone, methamphetamine; others
Condomless sex under the influence of drugs	Yes/no and which
Slam: parenteral drug use in the sexual context	Yes/no
Chemsex sessions: sexual relations with multiple people under the influence of drugs	Yes/no
Use of apps to search for sexual contacts in the year before HIV diagnosis	Yes/no

Cop: genome copies; GHB: gamma hydroxybutyrate; PEP: post-exposure prophylaxis; PrEP: pre-exposure prophylaxis; STI: sexually transmitted disease.

^a^ HIV recent seroconverters were considered to be those who had a documented negative HIV serology in the 12 months before diagnosis or who were in the process of seroconversion.

^b^ STI included *Neisseria gonorrheae* infection, *Chlamydia trachomatis* infection/lymphogranuloma venereum, syphilis, herpes simplex virus infection, anogenital condylomas, scabies, pediculosis pubis and hepatitis A, B and C.

We defined two categories of late presentation: late diagnosis with 350-500 cells/µL CD4^+^ T-cell count; and advanced disease with less than 200 cells/µL.

### Diagnostic techniques 

The detection of antibodies against HIV in serum was performed by fourth-generation chemiluminescence magnetic microparticle immunoassay (CMIA) (HIV 1/2 Ag Ab Architect Abbott Laboratories, Wiesbaden, Germany) which detects antibodies to HIV-1/HIV-2 and p24 antigen of HIV-1. A positive result was confirmed by Western blot (BIO-RAD, Marnes-la-Coquette, France) and Geenius HIV ½ Confirmatory Assay (BIO-RAD) techniques. The quantification of HIV plasma viral load was carried out through real-time PCR (Versant kPCR Siemens, Erlangen, Germany). After diagnosis, the CD4^+^  T-lymphocyte count was determined using CD45/CD3/CD8/CD4 flow cytometry (Epics XL and Aquios C.L. Beckman Coulter, Brea, US).

### Statistical analysis

The qualitative variables are presented as absolute and relative frequencies. The quantitative variables are expressed as the mean and standard deviation (SD). No data imputation has been made, all denominators for calculations were based on available information. Unlike for the other variables, information on chemsex and apps were only collected from 2016 to 2019. Comparison of the trend of the qualitative variables of the new HIV diagnoses based on years was carried out with the chi-squared test for linear trend and the quantitative variables by the analysis of variance (ANOVA). The chi-squared for trend calculated was based on the Mantel-Haenzzel (MH) statistic. This test, being based on a linear function of frequencies, is less demanding than Pearson's chi-square test. It can be applied with expected frequencies greater than 2. In all comparisons, the application conditions were met, except for the age variable. In that case, the ranges 50–59 and ≥ 60 have been unified. For all tests, we accepted a significance value of 5%. The statistical analysis of the data was carried out using the STATA 15.0 statistical package (StataCorp LLC, College Station, US).

### Ethical statement 

All data derived from medical histories were fully anonymised before access. The study protocol was approved by the IRB of Hospital Clínico San Carlos, approval number 20/795-E. The ethics committee waived the need for informed consent because the information obtained for the study was collected in routine clinical practice.

## Results

**Figure 1:**
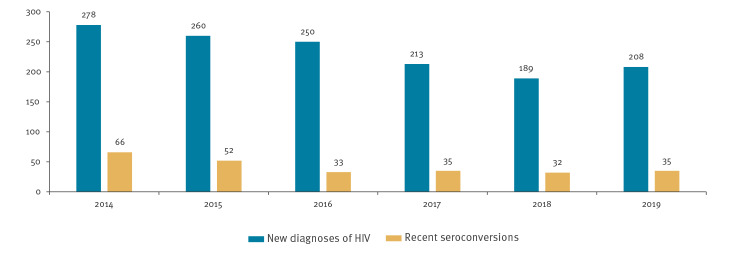
New diagnoses of HIV infection among men who have sex with men (n = 1,398) and cases of recent seroconversion (n = 253) documented at one reference centre, Madrid, 2014–2019

Between 2014 and 2019, 1,398 new infections of HIV were diagnosed among MSM. For 253 of them, a recent seroconversion could be documented. During that period, we observed a downward trend in the number of cases and of recent seroconverters (rSCV) (p = 0.034) (Figure 1).

### Sociodemographic and clinical characteristics of men who have sex with men and who had newly diagnosed HIV infection 

**Figure 2:**
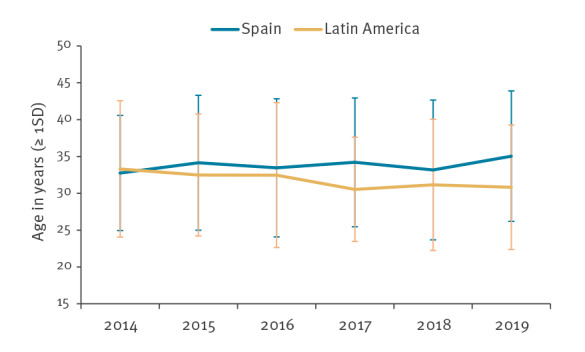
Average age at the time of HIV diagnosis, by place of origin (Spain or Latin America) among men who have sex with men, Madrid, 2014–2019 (n = 1,398)

The most relevant characteristics of MSM with new HIV diagnoses are described by year in Table 2, from 2014 to 2019. Most transmissions (97.9%) were sexual and 2.1% occurred in people who inject drugs (PWID). Intravenous drugs were used in a sexual context by all 2.1%. This practice, known as slam, remains infrequent but has increased during the study period. The average age was 32.9 years (± 8.8), ranging from 15.6 to 74.9 years. Diagnoses decreased among people born in Spain and increased among those born in Latin America, with a statistically significant trend (p linear trend < 0.001). Diagnosis of HIV occurred at a younger age among Latin American compared with Spanish MSM, with a significant trend (p linear trend < 0.001) (Figure 2). The majority were early diagnoses but, starting in 2016, the number of late diagnoses and advanced disease increased with a statistically significant upward trend.

**Table 2:** Most relevant sociodemographic and clinical characteristics of newly HIV-diagnosed men who have sex with men, 2014–2019 (n = 1,398)

Characteristics	2014 n = 278		2015 n = 260		2016 n = 250		2017 n = 213		2018 n = 189		2019 n = 208		Total n = 1,398		p (linear trend)
	%	n	%	n	%	n	%	n	%	n	%	n	%	n	
Mode of transmission															
Sexual	99.6	277	99.2	258	98.8	247	96.2	205	96.8	183	95.7	199	97.9	1,369	**< 0.001**
PWID/slam	0.4	1	0.8	2	1.2	3	3.8	8	3.2	6	4.3	9	2.1	29	
Age (years)															
≤ 19	1.4	4	0.4	1	1.2	3	1.9	4	1.6	3	0	0	1.1	15	0.151
20–29	40.6	113	44.2	115	41.2	103	43.7	93	49.7	94	47.6	99	44.1	617	
30–39	38.5	107	35.8	93	33.6	84	39.9	85	29.6	56	37.0	77	35.9	502	
40–49	15.5	43	15.0	39	19.2	48	10.8	23	12.7	24	10.6	22	14.2	199	
≥ 50	4.0	11	4.6	12	4.8	12	3.8	8	6.3	12	4.8	10	4.6	65	
Place of birth															
Spain	68.0	189	57.7	150	54.4	136	47.9	102	40.2	76	36.1	75	51.8	724	< 0.001
Latin America	25.2	70	31.9	83	36.0	90	42.3	90	55.0	104	58.7	122	40.0	559	
Other	8.3	23	10.4	27	9.6	24	9.9	21	4.8	9	5.3	11	8.2	115	
CD4^+^ T-cell count															
≤ 200	6.2	15	6.0	13	5.4	11	11.9	23	11.6	19	9.2	17	8.1	98	< 0.001
201–350	12.0	29	15.6	34	12.8	26	21.2	41	22.6	37	27.6	51	18.1	218	
351–500	16.2	39	22.5	49	23.6	48	25.9	50	31.7	52	25.4	47	23.7	285	
> 500	65.6	158	56.0	122	58.1	118	40.9	79	34.1	56	37.8	70	50.1	603	
Unknown	NA	37	NA	42	NA	47	NA	20	NA	25	NA	23	NA	194	
HIV viral load															
< 37	0.4	1	1.8	4	2.0	4	3.1	6	1.8	3	3.2	6	2.0	24	0.829
37–1,000	2.9	7	7.3	16	4.9	10	6.2	12	6.1	10	5.9	11	5.5	66	
1,001–10,000	17.4	42	10.6	23	14.3	29	17.1	33	17.1	28	14.1	26	15.0	181	
10,001–50,000	29.0	70	32.1	70	30.5	62	32.1	62	22.6	37	22.7	42	28.5	343	
50,001–100,000	17.0	41	13.3	29	20.7	42	15.0	29	15.9	26	11.9	22	15.7	189	
> 100,000	33.2	80	34.9	76	27.6	56	26.4	51	36.6	60	42.2	78	33.3	401	
Unknown	NA	37	NA	42	NA	47	NA	20	NA	25	NA	23	NA	194	
STI															
History of STI	70.9	197	62.3	162	71.6	179	69.0	147	70.4	133	76.0	158	69.8	976	0.074
Concomitant STI	48.9	136	53.1	138	60.8	152	56.8	121	63.5	120	68.3	142	57.9	809	< 0.001

NA: not applicable; PWID: people who inject drugs; STI: sexually transmitted infections.

No data imputation has been made, all denominators for calculations were based on available information (excluding those with missing information).

SD: standard deviation.

### Behavioural characteristics of men who have sex with men and who were newly diagnosed with HIV infection

The majority of newly diagnosed MSM began sexual relations at an age between 16 and 18 years, had more than 10 sexual partners in the year before diagnosis and more than 100 sexual partners in their lifetime. Consistent condom use was 0.4% (5/1,295) in oral sex, 13.6% (176/1,295) in insertive anal sex and 19.4% (251/1,295) in receptive anal sex. From the total, 69.8% had a history of STI and 57.9% had concomitant STI at the time of the HIV diagnosis. The request of pharmacological prevention was low, 3.8% had used PPE on some occasion and 1.1% had used PrEP. Use of alcohol 'in excess' or recreational drugs were recorded in 83.4%, and 64.5% had condomless sex under their effects. Chemsex sessions were noted in 21.4%, and 2.1% practiced slam (Table 3). Prior negative HIV test results were seen in 86.6% and recent seroconversion could be documented in 18.1%. The 81.1% (697/860) use apps for sex.

**Table 3:** Most relevant behavioural characteristics of newly HIV-diagnosed men who have sex with men, 2014–2019 (n = 1,398)

Characteristics	2014 n = 278		2015 n = 260		2016 n = 250		2017 n = 213		2018 n = 189		2019 n = 208		Total n = 1,398		p (linear trend)
	%	n	%	n	%	n	%	n	%	n	%	n	%	n	
Age at sexual debut (years)															
< 13	5.4	15	5.8	15	3.2	8	3.8	8	4.2	8	7.7	16	5.0	70	0.141
13–15	23.0	64	21.5	56	24.0	60	22.5	48	25.9	49	24.5	51	23.5	328	
16–18	46.0	128	45.4	118	46.0	115	48.8	104	49.2	93	46.2	96	46.8	654	
19–25	24.5	68	24.2	63	24.8	62	23.0	49	19.0	36	19.7	41	22.8	319	
> 25	1.1	3	3.1	8	2.0	5	1.9	4	1.6	3	1.9	4	1.9	27	
Number of sexual partners in lifetime															
1–10	7.2	20	5.1	13	6.0	15	5.2	11	18.5	15	6.3	13	6.3	87	0.271
11–100	44.6	123	37.8	96	34.5	86	33.8	72	39.8	70	39.3	81	38.4	528	
101–1,000	41.7	115	47.2	120	48.2	120	48.4	103	44.3	78	42.7	88	45.4	624	
> 1,000	6.5	18	9.8	25	11.2	28	12.7	27	7.4	13	11.7	24	9.8	135	
Unknown	NA	2	NA	6	NA	1	NA	0	NA	13	NA	2	NA	24	
Number of sexual partners in the year before diagnosis															
0–5	29.3	81	28.0	71	28.9	72	25.4	54	28.6	53	30.8	64	28.5	395	0.775
6–25	44.9	124	42.1	107	36.9	92	38.0	81	40.5	75	35.1	73	39.9	552	
26–100	12.0	33	13.4	34	17.3	43	28.6	61	19.5	36	22.1	46	18.3	253	
> 100	13.8	38	16.5	42	16.9	42	8.0	17	11.4	21	12.0	25	13.4	185	
Unknown	NA	2	NA	6	NA	1	NA	0	NA	4	NA	0	NA	13	
Sexual behaviour															
Sex worker	6.8	19	8.8	23	9.2	23	10.3	22	11.1	21	10.1	21	9.2	129	0.115
Use of drugs for sex	86.0	239	84.2	219	87.2	218	83.1	177	78.8	149	78.8	164	83.4	1,166	0.008
Condomless sex under effect of drugs	67.3	187	65.8	171	66.4	166	67.6	144	56.6	107	61.1	127	64.5	902	0.038
Chemsex sessions	Unk		Unk		22.0	55	19.2	41	16.9	32	26.9	56	21.4	184	0.343
Apps for sex	Unk		Unk		82.0	205	79.3	169	80.4	152	83.0	171	697	81.1	0.483

NA: not applicable; Unk: unknown.

No data imputation has been made, all denominators for calculations were based on available information (excluding those with missing information).

### Sociodemographic, clinical and behavioural characteristics among men who have sex with men and with recent HIV seroconversion

**Figure 3:**
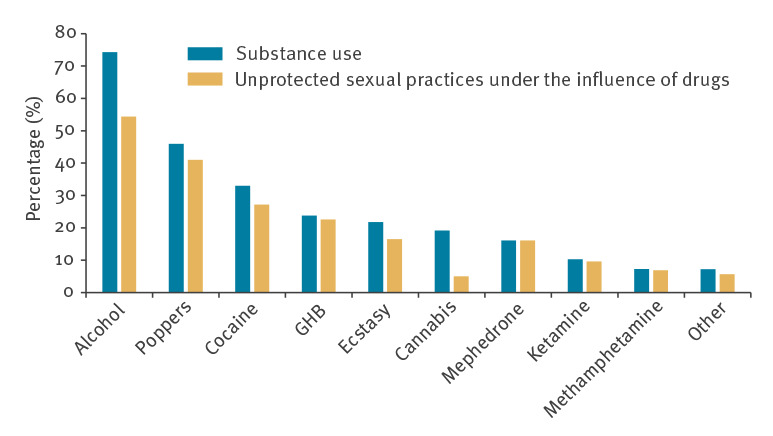
Frequency of condomless sexual practices under the effect of drugs, by substance type, recently seroconverted HIV-positive men who have sex with men, Madrid, 2014–2019 (n = 253)

**Figure 4:**
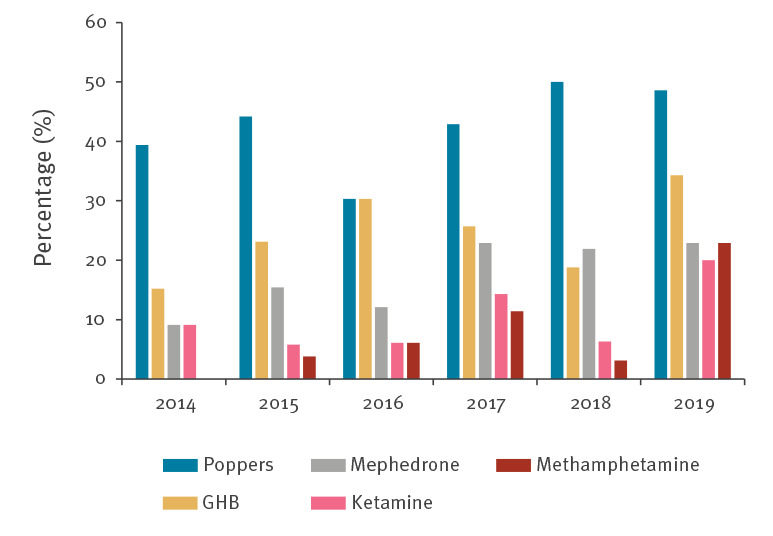
Frequency of use of substances associated with condomless sex in recently seroconverted HIV-positive men who have sex with men, Madrid, 2014–2019 (n = 253)

Among the 253 HIV rSCV, 80.2% (n = 203) were between 20 and 39 years-old; 65.6% (n = 166) were of Spanish and 26.5% (n = 67) of Latin American origin. Alcohol 'in excess' or recreational drugs were consumed on a regular basis by 88.1% (n = 223), and 73.9% (n = 187) had condomless sexual relations under their effects. The substances that were most associated with sex and having sexual relations without a condom were: mephedrone (n = 41; 100%), gamma hydroxybutyrate (GHB) (n = 59; 95.2%), methamphetamine (n = 17; 94.4%), ketamine (n = 28; 93.3%) and poppers (n = 107; 89.2%) (Figure 3). Use of some of these substances increased during the study period, particularly methamphetamine (Figure 4). Of the rSCV, 65 (28.9%) participated in chemsex sessions with an upward trend, eight (24.2%) in 2016, 10 (28.6%) in 2017, eight (25.0%) in 2018 and 13 (37.8%) in 2019. Apps were used to search for sexual contacts by 207 (92.6%) of the rSCV, with a stable trend during the period studied. The most common were: Grindr (n = 171; 83.0%), Scruff (n = 61; 29.6%) and Wapo (n = 48; 23.0%). Transmission of the infection was attributed to a sexual contact found through apps by 157 (70.4%). 

GHB: gamma hydroxybutyrate.

Substance use: Alcohol: 75.1% (190/253); poppers: 47.4% (120/253); cocaine: 33.2% (84/253); GHB: 24.5% (62/253); ecstasy 22.1% (56/253); cannabis: 19.4% (49/253); mephedrone 16.2 (41/253); ketamine 11.9% (30/253); methamphetamine: 7.1% (18/253) and others: 6.3% (16/253). Unprotected sexual practices under the influence of drugs: Alcohol: 46.2% (117/253); poppers: 42.3% (107/253); cocaine: 27.3% (69/253); GHB: 23.3% (59/253); ecstasy 16.6% (42/253); cannabis: 4.7% (12/253); mephedrone 16.2 (41/253); ketamine 11.1% (28/253); methamphetamine: 6.7% (17/253) and others: 4.7% (12/253).

GHB: gamma hydroxybutyrate.

Totals are n =66 in 2014; n=52 in 2015; n=33 in 2016; n=35 in 2017; n=32 in 2018; n=35 in 2019.

## Discussion

In our study, the percentage of people born in Latin America was higher and presented an upward trend, 25.2% in 2014 and 58.7% in 2019. In addition, the new HIV diagnoses among MSM born in Spain decreased significantly in our study, from 66.5% in 2014 to 36.1% in 2019, a more pronounced change than seen in the national figures [20]. In Spain, the number of new HIV diagnoses remains high among MSM, with a slower decline than in surrounding countries [15,19]. One of the reasons is the large number of cases originating from other countries with a high incidence of HIV infection, particularly from Latin America. According to the SINIVIH registry, 63.9% of all newly diagnosed people in 2019 were born in Spain and 22.9% Latin America [20]. Our data match with those recorded in San Francisco where, from 2012 to 2018, the percentage of new HIV cases decreased significantly among white people and increased among African and Latino Americans [17]. The group with most new HIV cases in San Francisco changed from white to Latino between 2017 and 2018, while we saw a similar change in our work between 2018 and 2019.

In high-income countries, a downward trend in new HIV diagnoses has been observed, especially since 2016, and for the first time in the history of the HIV epidemic, also among MSM. This trend has been even more noteworthy in cities where a combination of HIV prevention strategies have been implemented with the inclusion of PrEP [17,18]. According to data published by the United Kingdom’s (UK) National Health Service, new HIV diagnoses went down by 34% globally from 2015 to 2019 [21]: The reported decline among MSM was significative, from a peak of 3,214 in 2014 and 2,079 in 2018 to 1,700 diagnosed in 2019 (a 47% and 18% drop, respectively). The steepest declines were observed among MSM of white ethnicity (2,550 in 2014, 1,425 in 2018 and 1,107 in 2019) and MSM born in the UK (1,869 in 2014, 950 in 2018 and 715 in 2019) [21]. In our study, the new HIV infections decreased more slowly. Other parts of the world, for example in Latin American countries, have experienced an increase in the incidence of HIV, the number of new infections in that region increased by 7% between 2010 and 2018 [15].

In our study, 47.6% of MSM diagnosed in 2019 were between 20 and 29 years-old and 37.0% between 30 and 39 years, compared with the national SINIVIH data where 26.9% newly diagnosed people were between 20 and 29 and 32.3% between 30 and 39 years-old [20]. The majority of new HIV diagnoses in Europe occur in people between 20 and 39 years of age, with a downward trend in all age groups, except in those older than 50 years, where it remains stable [19]. According to our temporal analysis, increasingly younger people were diagnosed with HIV. This trend was linear and significant among Latin Americans, whereas among Spanish people it remained stable at around 33 years of age.

If we compare the CD4^+^ T-lymphocyte counts at the time of HIV diagnosis between the Spanish national registry against in our study among MSM they were, in 2019, 378 vs 533 cells/mL on average. There were 20.5% vs 9.2% of diagnoses with < 200 cells/mL advanced disease and 20.8% vs 27.6% with 201–350 cells/mL late diagnosis [20]. This difference between the data from Madrid and the governmental data reflect an earlier diagnosis in our STI/HIV clinic, probably related to the follow-up of seronegative people with risky practices. However, in the years 2017 to 2019, the CD4^+^ T-cell counts at the time of diagnosis have been lower. This may be explained by later diagnosis among foreigners and the numerous diagnoses of acute infection, coinciding with the decrease in CD4^+^ T-cells.

The WHO points out that MSM are highly vulnerable to acquiring HIV infection [22]. In this work, MSM presented clinical and behavioural markers considered to indicate high risk: history of STI (69.8%), high number of sexual partners, condomless sexual practices under the effect of recreational substances (64.5%) and the use of apps to search for sexual contacts (77.4%). Several publications have associated the use of recreational drugs with an increased incidence of HIV and other STI [23]. Among the MSM analysed, 64.5% had condomless sexual relations under the influence of drugs, while among the rSCV, this percentage was higher (73.9%) and similar to that found in a meta-analysis carried out among HIV-positive people and including 38 publications from 2000 to 2018 [24]. The USEX-Study, a study conducted in 22 hospitals in Madrid with 2,916 HIV-positive MSM, found that 29.1% used drugs for sex and, in our study, 28.9% of the rSCV participated in chemsex sessions [25]. It is fundamental to know the type of drug used and the route of administration. Substances such as mephedrone, methamphetamine, GHB, ketamine and poppers were strongly related to condomless sex and, as Pakianathan et al. noted, were identified as facilitating drugs for HIV transmission [26]. Even though their use by the parenteral route is uncommon, it is increasing, from 0.4% in 2014 to 4.3% in 2019. All PWID In our study used substances in a sexual context, data which are in line with Bui et al. who recorded that 4.7% of MSM monitored in an Australian Clinic practiced slam [27]. The growing popularity of chemsex coincides with a decrease in new HIV diagnoses, which could be explained by the high preventive efficacy of ART and PrEP. However, the use of preventive strategies such as PEP (3.8%) or PrEP (1.1%) was very rare in our study. The comparatively late funding in the implementation of PrEP in Spain could be one of the factors that explains the slow decline in new cases compared with other European or North American countries [14,28]. Funding of PrEP, combined with the other measures used in our country, may represent an opportunity to achieve a more substantial reduction in the number of new cases.

The use of apps to search for sexual contacts was high among MSM, especially among rSCV, and, as has been published elsewhere, the use of some social networks has been linked to risk behaviours for acquiring HIV and others STI [29]. In our work, 70.4% of HIV rSCV attributed the transmission of the virus to the use of these apps.

Our work could be limited by the fact that it was a monocentric study and analysed MSM, since most of the diagnoses in our centre were made among them. These data could not be extrapolated to heterosexual men and women or transgender women with very different sociodemographic, clinical and behavioural characteristics. It is an important line of research that should be studied. Despite this limitation, this is a study carried out at a reference STI/HIV clinic where care is offered without administrative barriers. Half of the people attended to are MSM, and many of them are of foreign origin, mainly Latin American. Because of the fact that it is the reference centre for PrEP in the Community of Madrid and because of its particular characteristics, it is considered a sentinel centre prepared to detect the epidemiological changes that accompany an HIV epidemic at an early stage. Knowing the profile of people newly infected with HIV allows for the design of preventive strategies specifically aimed at each individual or population group.

## Conclusion

Our results reflect a reduction in new cases of HIV, in particular among MSM born in Spain, although the trend is not as significant as it is in other countries. Combination of HIV prevention strategies with the inclusion of PrEP and frequent HIV testing, should be reinforced among young MSM, especially those born in Latin America, those that use drugs for sex and those that use apps in search of sexual contacts.
